# Improving the Management of Late-Life Depression in Primary Care: Barriers and Facilitators

**DOI:** 10.1155/2011/326307

**Published:** 2011-05-05

**Authors:** Tamara Sussman, Mark Yaffe, Jane McCusker, David Parry, Maida Sewitch, Lisa Van Bussel, Ilyan Ferrer

**Affiliations:** ^1^School of Social Work, McGill University, 3506 University Street, Room 300, Montreal, QC, Canada H3A 2A7; ^2^Family Medicine, St. Mary's Hospital Center, McGill University, 3830 Lacombe Avenue, Montreal, QC, Canada H3T 1M5; ^3^Epidemiology, Biostatistics and Occupational Health, St. Mary's Hospital Center, McGill University, 3830 Lacombe Avenue, Montreal, QC, Canada H3T 1M5; ^4^Department of Clinical Epidemiology and Community Studies, St. Mary's Hospital Center, Faculty of Law, McGill University, 3830 Lacombe Avenue, Montreal, QC, Canada H3T 1M5; ^5^Department of Medicine, McGill University, 3830 Lacombe Avenue, Montreal, QC, Canada H3T 1M5; ^6^Division of Geriatric Psychiatry, Department of Psychiatry, St. Joseph's Health Centre, The University of Western Ontario, 850 Highbury Avenue, London, ON, Canada N6A 4G5; ^7^School of Social Work, McGill University, 3506 University Street, Montreal, QC, Canada H3A 2A7

## Abstract

The objectives of this study were to elicit Canadian health professionals' views on the barriers to identifying and treating late-life depression in primary care settings and on the solutions felt to be most important and feasible to implement. A consensus development process was used to generate, rank, and discuss solutions. Twenty-three health professionals participated in the consensus process. Results were analysed using quantitative and qualitative methods. Participants generated 12 solutions. One solution, developing mechanisms to increase family physicians' awareness of resources, was highly ranked for importance and feasibility by most participants. Another solution, providing family physicians with direct mental health support, was highly ranked as important but not as feasible by most participants. Deliberations emphasized the importance of case specific, as needed support based on the principles of shared care. The results suggest that practitioners highly value collaborative care but question the feasibility of implementing these principles in current Canadian primary care contexts.

## 1. Introduction

Clinically significant depression is prevalent among 10–25% of older community-dwelling adults [1–3]. Although some patients avoid disclosing mental health issues to family physicians, the majority of older adults with depression prefer to seek treatment in general health care settings such as family practices rather than psychiatric settings [4–6]. Perhaps as a consequence, depression is the most common late-life mental health disorder to present in primary care [7]. Late-life depression can be successfully treated with antidepressant medication and/or psychotherapy. However, to date, few depressed older adults receive these treatments in primary care [8, 9]. This compromises older adults' health, quality of life, and physical functioning [10] and increases their risk of mortality [5, 6], negatively impacts the health of their families [11], and augments health care costs [12, 13]. 

Despite the documented underidentification and undertreatment of depression in primary care, many family physicians are comfortable managing adult depression and see it as their role to attend to common mental health issues such as depression and anxiety [14–16]. This suggests that organizational barriers such as limited time, remuneration formulas, and poor links between family practices and mental health teams may contribute to current levels of undertreatment and underdetection [17].

The present study aimed to elicit Canadian health professionals' views on the barriers to identifying and treating late-life depression in primary care settings and on the solutions felt to be most important and feasible to implement. We present the results of a consensus development process that included a conference attended by an interdisciplinary group of practitioners interested in late-life depression. The conference and post-conference deliberations were informed by an integrated knowledge translation approach that engaged those in a position to act on research, that is, knowledge users, into the “front end” of the research process [18, 19]. Three primary questions informed conference/post-conference deliberations and analyses: (1) what do practitioners from a variety of professional backgrounds think interferes with the identification and management of late-life depression within primary care, (2) what solutions do they think might address these barriers, and (3) what might practitioners need to know to facilitate the implementation of the solutions they considered to be most important?

## 2. Methods: Preconference Activities

A snowballing process was used to invite practitioners from two Canadian provinces (Ontario and Quebec) for participation in a 1-day conference held in Montreal, Quebec on December 2nd, 2008. This purposeful approach to sampling was used to ensure that invited participants had an interest in late-life depression and represented a variety of health disciplines and practice contexts. Potential participants known to the research team were solicited by telephone for potential participation and for the identification of additional potential participants. Individuals identified by others were sent a letter of invitation followed by a telephone call from the project coordinator. Twenty-five potential participants were identified through this method, with 12 (3 family physicians, 2 psychiatrists, 3 nurse practitioners, 3 social workers, and one decision-maker) being available to attend. Of the 12 researchers, eight (3 family physicians, 1 psychiatrist, 1 social worker, 2 epidemiologists, and 1 social science researcher) were also able to attend. Travel or displacements costs for participants were covered by the study.

The research was conducted in accordance with the standards of the* Tri-Council Policy Statement for Ethical Conduct for Research Involving Humans* [20] as required by all researchers in Canadian universities. Procedures were approved by the St. Mary's Hospital Centre Ethics Board.

## 3. Methods: Conference Activities

The conference program was composed of a combination of plenary and small group break-out sessions. The morning plenary provided a review of the preliminary evidence supporting the effectiveness of managing late-life depression in primary care, using the chronic care model [21–24] to structure the discussion, and offering an opportunity to reflect on collaborative care principles. 

The plenary was followed by a structured brainstorming session conducted in small break-out groups. The break-out discussions asked participants to consider barriers and solutions related to (1) identifying depressed older patients, (2) engaging older adults in the treatment of late-life depression, and (3) improving collaboration between primary care clinicians and mental health clinicians. Participants were preassigned to one of three groups. All break-out groups included a combination of primary care physicians, psychiatrists, nurses, and social workers. Each group was facilitated by two members of the research team. The researchers, most of whom were also clinicians, actively participated in group discussions.

An adapted nominal group technique was used to structure the break-out group discussions. This guided brainstorming technique is an effective idea-generating technique that facilitates the equal expression of ideas and equitable decision-making processes among participants [20]. 

In the afternoon plenary, each group provided a summary of its discussions focusing on identified barriers and prioritized solutions. Feasibility and acceptability of proposed solutions were then discussed in depth within the plenary.

## 4. Methods: Post-Conference Activities

The final stage of a nominal group technique involves the ranking of proposed ideas. In its original form, the generation of ideas, subsequent discussion, and ranking are undertaken at one meeting. In our modified process, conference participants were sent a final list of solutions via e-mail one week following the conference. Participants ranked each idea twice, once according to importance and once according to feasibility. Voting post-conference gave participants additional time to reflect on the relative importance and feasibility of each proposed solution. Eleven invited conferences participants and the 5 researchers who were direct practitioners ranked solutions. Most nonrespondents indicated that other demands precluded them from submitting their final votes within the requested time frame. 

The list of solutions generated at the conference was circulated to an additional group of practitioners, who did not participate in the conference. These post-conference participants were purposefully selected to represent practitioners' experiences within other provinces (Alberta and British Columbia), and experts involved in the development of local, provincial, or national mental health reforms. Of the 24 invited post-conference participants, seven were able to provide feedback. The seven participants included 2 family physicians, 2 psychiatrists, 2 administrative directors of mental health programs, and 1 nurse. Three of these participants were involved in the development of mental health reforms.

Post-conference participants were sent the list of proposed solutions generated at the conference and asked to rank them for importance and feasibility. Participants were further asked to comment in writing or by telephone on the comprehensiveness of the proposed solutions.

## 5. Methods: Analysis of Data

Each generated solution was rated for importance and feasibility using two methods: (1) mean importance and (2) feasibility frequency which tallied the number of times a solution was ranked as either a first, second, or third choice. Conference (*n* = 16) and post-conference ratings (*n* = 7) were kept separate. Post-conference comments related to the comprehensiveness of proposed solutions were recoded dichotomously as either comprehensive and complete (1) or not comprehensive and complete (0). 

All small group conference discussions were tape recorded and transcribed verbatim. A three-step process was used to analyze the transcripts [23]. In step one, text segments from each small group were examined and given a preliminary code using specific utterances from the text. In step two, specific utterances were linked together into categories that shared common factors. In the final step, categories were linked to broader themes repeated both within and across small groups. One investigator (TS) and one research assistant (IF) worked independently and then together at each stage of the data analysis process. Other members of the research team reviewed the broad themes for accuracy. Only themes upon which there was agreement were used in the final analysis. Data were analyzed using QSR's International NVivo 8 [25]. 

## 6. Results: Quantitative Findings

Conference attendees generated 12 solutions (Table 1). Conference and post-conference attendees' rankings are presented in Tables 1 and 2, respectively. According to frequency rankings, two solutions were identified as most important by both groups: (1) providing family physicians with direct mental health support for complex cases and (2) developing mechanisms to increase family physicians' awareness of resources to help them manage depression. Both solutions were endorsed by 44% of conference and 57% of post-conference participants. Providing direct mental health support was ranked as the most important solution (#1) by all 57% of the post-conference participants that endorsed it. According to mean rankings for conference participants, allocating monetary and human resources towards facilitating collaborative care was also seen as an important solution and was ranked amongst the top three important solutions by 38% of conference participants. This solution was not ranked as highly by post-conference participants.

According to frequency rankings, two solutions were seen as most feasible by both groups; (1) developing mechanisms to increase family physicians' awareness of resources to help them manage depression and (2) provision of general education on depression. Developing mechanisms to provide family physicians with direct support from mental health professions, tied for first place as most important solution for both groups, was only endorsed by 19% of conference participants and 29% of post-conference participants for feasibility. The mean ranking for post-conference participants, however, placed direct mental health support among the top two feasible solutions.

Allocating monetary and human resources to make collaborative care easier to engage in was only endorsed by 6% of conference participants for feasibility. No post-conference participants endorsed this solution for feasibility.

Six of the seven post-conference participants considered the 12 generated solutions to be complete and comprehensive. The participant who did not consider the solutions to be complete commented that the solutions were narrowly focused on medical practitioners.

## 7. Results: Qualitative Findings for Conference Attendees

Although the three work groups were asked to address slightly different aspects of identification and treatment, particular barriers and solutions were repeatedly highlighted in all groups. These convergent themes represent another form of data capturing the perceived importance and feasibility of solutions.

### 7.1. Comorbidity

According to conference participants, older adults living with depression are rarely “just presenting depression” (family physician). More typically older adults present with a series of physical complaints some of which may be associated with other comorbid conditions. As one family physician said “(an older person) will not arrive in your office and say “I am depressed” he/she will present a series of somatic complaints.” This presentation complicates the identification and treatment of depression as both the physician and patient may be more inclined to prioritize other conditions. With “the competing priorities of other conditions” (family physician), participants emphasized that working with late-life depression requires complex problem solving, decision making, and time.

### 7.2. Silo Mentality

Participants repeatedly noted the discrepancy between the clinical realities of managing depression, and the “silo” nature of health delivery systems which supports the notion of specialized teams “all of whom will help you separately” (family physician). According to participants, this silo *system* can foster a silo *mentality* where service providers within the system refrain from sharing information and decision-making across teams. As one Quebec-based family physician said “family doctors refer to a black box and don't hear much back on their patients … there's a clear silo mentality in the … system”. A nurse participant suggested that having separate mental health teams allows family physicians to exercise a hand off mentality with the attitude “I'm referring him, please take care of this patient.” She continued that this type of sentiment is not seen as readily in other chronic conditions. As she stated “it's rare that you will see that in cardiology, if your patient has an unstable angina, you will ask for the cardiology evaluation … and the patient will come back to you and you will manage him”.

### 7.3. Developing Collaboration through Case-Specific Support

Given the clinical complexities involved in identifying and treating late-life depression and the silo attitude of many practitioners and teams within the mental health system, participants felt that strengthening true collaboration between family physicians and mental health teams was key to improving the management of late-life depression. While participants offered a variety of solutions including infusing the medical curriculum with content on collaboration, offering interdisciplinary skill-based training to build collaboration, improving funding formulas to account for time necessary for collaboration, and working with organizations to develop better mechanisms to share information, most participants emphasized that their best collaborations evolved on a case by case basis as they found health professionals on mental health teams with whom they could readily consult, solve problem, and share information. As one family physician stated “we used to have a nurse practitioner linked to the psycho-geriatric team here at the hospital who used to have a particular interest in making herself available to family doctors who would call in and say “I do not need to have to see your psychiatrist but I do need to know about what fits with this patient at this time” … and she was outstanding.”

### 7.4. Case-Specific Support Offered “Just in Time”

Participants suggested that the most effective method of supporting family physicians in the management and treatment of late-life depression was to provide them with access to an individual (in person or on the phone) with whom they could consult as needed. As one family physician said “when I have a problem I need a solution and a resource to solve that problem right then and there. I may not remember all of the information presented to me at one time or another. (It would help) if I had a resource person to call to inform me of what is available at the time that I need it … information just in time.” 

Participants suggested that family physicians could benefit from two types of “just in time” support when identifying and managing late-life depression. First, family physicians could benefit from decisional support for the treatment of depression in complex cases (e.g., where comorbidity exists). Second family physicians could benefit from informational support such as specific mandates of specialized teams and of community programs that can support older adults living with depression. Decisional support in depression management for complex cases was seen as critical to increasing physician confidence in the identification and treatment of late-life depression. Accessible and pertinent information about resources was highlighted as instrumental to the facilitation of collaboration as “you can't collaborate if you do not know who to collaborate with” (family physician).

Initiatives such as fact sheets of community resources and algorithms of stepped approaches to depression treatment were offered as possible mechanisms to address the need for decisional support around treatment and linking patients to resources. Some participants questioned the relevance of these static approaches, suggesting that such information was not necessarily applicable to the cases seen in practice, and that these guides were often lost or forgotten when needed.

## 8. Discussion

We sought to elicit Canadian providers' views on the barriers to identifying and treating late-life depression in primary care settings and the solutions felt to be most important and feasible. These objectives were addressed through a consensus process that included a conference attended by an interdisciplinary group of practitioners with an interest in late-life depression, and post-conference consensus development methods.

 Most conference and post-conference participants thought that the provision of direct mental health support to family physicians for complex cases and the provision of mechanisms to increase family physicians' awareness of resources were key to addressing current barriers to the identification and treatment of late-life depression. Conference attendees emphasized through their deliberations that the mental health and resource support family physicians required had to be (1) case specific, (2) provided by a trustworthy professional who was knowledgeable with mental health treatment and services, and (3) available as needed. Passive forms of decisional support (such as clinical guidelines, algorithms, and information systems that post follow-up reminders to clinicians) could supplement but not replace the personalized support offered by another professional. Indeed, research suggests that static forms of decisional support are perceived as difficult to implement, especially in complex clinical cases where comorbid illnesses complicate medical decision making and management [26]. Further, static support systems are rarely effective in improving patient outcomes unless accompanied by organizational support to enhance patient care [27]. The most effective enhancements appear to be (1) the presence of a case manager with a mental health background augmenting the care provided by a primary care physician and (2) regular psychiatric supervision [28]. 

The conference deliberations further emphasized that participants thought true collaboration involved the sharing of case information, treatment decisions, and follow-up amongst team members. While the extent of collaboration between family physicians and mental health teams has not been shown to independently improve clinical outcomes in patients, findings from this conference and elsewhere suggest that family physicians, psychiatrists, social workers, and nurses may prefer a shared care model of collaboration to parallel/hand off approaches to care [28]. In this way, collaborative care models that involve collective decision making are more likely to be acceptable to practitioners. 

Conference deliberations suggested that the key to improving collaborative attitudes was to offer collaborative* experiences* where through involvement and discussions of case material, professionals could begin to collectively negotiate roles and responsibilities. Seen in this regard, collaboration cannot be as effectively taught through skills-based training and curriculum content but rather is best learned through experience. 

Collaborative care models that involve the co-location of mental health professionals within family practice have shown that these experiences do contribute to case sharing and collective decision-making over time. They also contribute to professional satisfaction [29, 30]. This outcome appears to be particularly important in the area of mental health where family physicians and mental health specialists have expressed dissatisfaction with the one another's contributions to the identification, management, and treatment of mental health cases [31]. Fostering opportunities for closer collaboration likely allows professionals to negotiate expectations with one another thereby potentially reducing dissatisfaction and encouraging further collaboration.

While conference and post-conference participants placed a high degree of value on the provision of direct mental health support to family physicians, they did not think that these collaborative care components could be feasibly implemented in their contexts. This suggests that one of the most important components of collaborative care to participants was not seen to be feasible to implement within their current contexts. While the mean rank of post-conference participants suggested a slightly more optimistic view on the feasibility of direct mental health support, tallied rankings confirmed that most post-conference attendees also questioned the feasibility of this solution. 

The collaborative care models currently developed and implemented in Canada that involve the components valued by conference attendees include a shift from fee for service care to alternate payment programs based on capitation. Currently, about 40% of family practitioners in Canada are still solo practitioners who bill directly to the state under a fee for service system [32]. Indeed many family physicians participating in this process were working within a fee for service context and most allied health professionals were collaborating with solo practitioners. It therefore remains important to locally develop and test the feasibility and sustainability of collaborative care models that include the provision of as needed direct mental health support and resource support to family physicians practicing in a variety of primary care contexts.

### 8.1. Study Limitations

Study limitations include the small, purposeful sample of health professionals with an interest in the management of late-life depression who may not be representative of the population of health providers in Canada. Decision makers were underrepresented in our group of conference and post-conference participants which may have impacted impressions about the feasibility of allocating human and monetary resources towards collaborative care initiatives.

## 9. Conclusion

Models of collaborative care that include provision of personalized support, as needed, to family physicians and that are based on shared care principles may be highly valued by family physicians, psychiatrists, nurses, and social workers in Canadian healthcare settings. Although components of collaborative care prioritized by this group were supported in the literature, study participants expressed apprehensions about the feasibility of implementing prioritized components of collaborative care in real world settings. This suggests that the “buy in” and confidence necessary for widespread adoption of collaborative care models are far from prevalent in Canadian primary care settings. Future research must address these concerns by engaging practitioners, decision makers, and researchers in the development and pilot testing of collaborative care models that include the prioritized components of collaborative care mentioned above and that are implemented in the variety of primary care settings currently represented in the Canadian primary care system.

## Figures and Tables

**Table 1:** Mean and frequency rankings of important and feasible solutions to depression management ranked by conference attendees (*N* = 16).

	Importance		Feasibility	
Solutions	Mean rank	Top three frequency	Mean rank	Top three frequency

Develop mechanisms to improve family physicians' awareness of resources to help manage depression	5.06	7	3.06	10
Develop mechanisms to provide family physicians with direct support from mental health professionals to help them manage specific patients	4.69	7	6.06	3
Monetary and human resources should be allocated to make collaborative care of depression easier to engage in	5.06	6	9.56	1
A framework to access depression care services from many settings should be developed	6.20	6	7.33	3
Improve coordination and flow of information between patients/families and physicians/health teams	6.22	5	5.88	5
Community-based resources should be enhanced to support older adults with depression and their families	6.19	4	6.19	2
Professional training on interdisciplinary collaboration in mental health should be provided	7.40	3	7.27	2
There should be increased lobbying efforts to secure funding	7.19	3	8.00	3
Computerized information systems should be implemented to foster better coordination—communication between family physician offices, hospital, and mental health teams	7.90	3	10.33	0
Case finding strategies should be implemented at strategic moments	6.97	3	5.19	5
Patients should be motivated, coached, and supported in their own self care efforts	7.00	1	4.88	5
General education on depression should be supplied	7.16	0	3.44	10

**Table 2:** Mean and frequency rankings of important and feasible solutions to depression management ranked by post-conference participants (*N* = 7).

	Importance		Feasibility	
Solutions	Mean rank	Top three frequency	Mean rank	Top three frequency

Develop mechanisms to improve family physicians' awareness of resources to help manage depression	4.57	4	4.43	4 (ranked 1 by all)
Develop mechanisms to provide family physicians with direct support from mental health professionals to help them manage specific patients	3.14	4 (ranked 1 by all)	5.29	2
Monetary and human resources should be allocated to make collaborative care of depression easier to engage in	6.42	2	10.43	0
A framework to access depression care services from many settings should be developed	6.14	0	6.86	1
Improve coordination and flow of information between patients/families and physicians/health teams	5.86	2	5.71	1
Community-based resources should be enhanced to support older adults with depression and their families	6.14	1	5.71	1
Professional training on interdisciplinary collaboration in mental health should be provided	6.71	2	6.00	2
There should be increased lobbying efforts to secure funding	7.71	0	8.14	0
Computerized information systems should be implemented to foster better coordination—communication between family physician offices, hospital, and mental health teams	10.00	0	10.71	0
Case finding strategies should be implemented at strategic moments	5.71	2	6.00	3
Patients should be motivated, coached, and supported in their own self care efforts	7.71	1	5.00	3
General education on depression should be supplied	5.43	3	3.71	4
